# Dr. Jacob F. Meyer

**Published:** 1902-07

**Authors:** 


### OBITUARY NOTES.

Dr. Jacob F. Meyer, of Buffalo, one of the most capable of the younger surgeons of this city, was killed in his office at 191 William Street, Friday evening, June 20, 1902, whether by design or accident has not transpired at this writing. While Dr. Meyer was attending to his patients during his evening hour, his wife hurriedly came into his office, remained a few minutes, hastened out, and soon afterward he was found dead on the floor from a pistol wound of the heart. Mrs. Meyer, who is in custody, says that she and her husband became engaged in a struggle for the possession of the pistol which exploded with the fatal results stated. Dr. Meyer was a graduate of Niagara University, class of 1891, afterward serving as interne at the Buffalo Hospital Sisters of Charity, and then several years as resident assistant at the Woman's Hospital. He developed an aptitude for surgery and is credited with having made many skilful operations with successful results. His sad end was a great shock to the community, and especially to his numerous immediate friends.

